# Correlation Between Objectively Measured Spontaneous Physical Activity and Sedentary Behavior With Frailty Syndrome in Older Adults: A Systematic Review and Meta‐Analysis of Observational Studies

**DOI:** 10.1155/jare/6085101

**Published:** 2026-05-31

**Authors:** Samuel Gonçalves Encarnação, William Alberto Latorre Martinez, María Martínez-Ferrán, Helios Pareja-Galeano

**Affiliations:** ^1^ Department of Physical Education, Sport and Human Movement, Universidad Autónoma de Madrid, Madrid, Spain, uam.es; ^2^ Advanced Research in Integrative Physiology for Life Research Group (IAFIV), Universidad de Burgos, Burgos, Spain, ubu.es

**Keywords:** accelerometer, frailty, lifestyle, physical inactivity

## Abstract

**Purpose:**

To analyze the correlation between spontaneous physical activity (PA) and sedentary behavior (SB) objectively measured using wearable devices and frailty syndrome (FS) in older adults.

**Methods:**

The PubMed/Medline, Web of Science, and Cochrane Library databases were used as sources of information. The search equation used was “(accelerometer OR physical activity) AND (elderly OR ‘older adult’ OR older) AND (frailty) AND (‘nursing home’ OR institutionalized).”

**Results:**

Twenty‐three studies were included. Of these, 14 studies examining PA were eligible for meta‐analysis. A random effects correlation analysis of these 14 studies revealed a significant inverse correlation between PA and FS (*r* = −0.48, 95% CI = [−0.5585, −0.3560], *Z* = −8.39, *p* < 0.0001), indicating statistically significant between‐study variance and high heterogeneity (*I* ^2^ = 84%). Regarding SB, 11 of the 16 studies were meta‐analyzed. Random‐effects meta‐analysis showed that 8 of the 11 analyzed studies presented a significant positive association between SB and FS (*r* = 0.334, CI = [0.2233; 0.4259], *Z* = 6.67, *p* < 0.0001), with statistically significant between‐study variance and high heterogeneity (*I* ^2^ = 86.9%). Sensitivity analysis revealed that removing one study increased the inverse correlation coefficient between PA and FS from −0.33 to −0.46 and increased the positive correlation coefficient between SB and FS from 0.22 to 0.39.

**Conclusion:**

Lower PA and high SB time are significantly associated with high FS incidence among older people. These findings highlight the importance of promoting PA and reducing SB to prevent FS and its complications in later life.

## 1. Introduction

Physiological aging leads to reduced organ function, diminished reserves, and disrupted homeostasis, contributing to the frailty syndrome (FS) [1]. FS is defined as a condition resulting from a reduction in the functional capacity of multiple systems. This increases the likelihood of disability and the need for supervision in activities of daily living (ADLs) [2]. Frailty is also considered a clinical state of vulnerability to stressors, characterized by an abnormal decline in physiological reserves and a reduced ability to maintain or restore body homeostasis [3]. Moreover, many frail older adults have other chronic conditions such as heart failure, diabetes, osteoporosis, osteoarthritis, pulmonary disease, or renal insufficiency [4].

However, FS can be reversed or attenuated by interventions that target the underlying causes of the syndrome [5]. Physical activity (PA) is key to preventing the physical and cognitive decline associated with FS [6]. Evidence shows that regular PA and reduced sedentary behavior (SB) are linked to a lower risk of chronic diseases, increased longevity, and improved physical and mental health. These habits can help to reduce or attenuate frailty in older adults [7, 8]. When 150 min of moderate‐intensity PA per week is not feasible due to chronic conditions, older adults should still be as active as their condition allows [9]. Conversely, prolonged SB increases visceral fat, inflammation, and the risk of conditions such as high cholesterol, hypertension, and diabetes. All of these factors contribute to frailty and negatively impact the health and well‐being of older adults [10].

Measuring PA is associated with certain challenges. A valid instrument must capture its multidimensional nature, including frequency, duration, and intensity levels. The most commonly used method for measuring PA levels is subjective questionnaires [11] because of their practicality and reduced cost. However, this method has limitations in terms of validity and reliability [12, 13]. Consequently, objectively quantifying these variables is necessary to better understand daily PA and SB and produce more reliable inferences. This is preferably achieved using wearable motion sensors such as accelerometers [14].

Systematic reviews using objective measurements from wearable devices have generally shown associations between spontaneous PA, SB, and frailty status in older adults [15–17]. However, an important gap remains in the literature: There is an inconsistent relationship between these behaviors and frailty status. While most literature indicates that frail individuals spend less time in moderate‐to‐vigorous PA and more time in sedentary activities [18], some studies have reported unexpectedly longer sedentary time for nonfrail individuals compared to their frail counterparts [19]. This lack of consensus underscores the need for the quantitative synthesis performed in this work, as no meta‐analyses have yet objectively summarized the relationship between spontaneous PA, SB, and frailty in older adults. Therefore, this systematic study aimed to analyze the correlation between spontaneous PA and SB objectively measured using wearable devices and FS in older adults. We hypothesized that higher levels of spontaneous PA (without specific exercise training programs) and reduced SB would be associated with a lower risk of frailty in older adults.

## 2. Material and Methods

This systematic review was conducted in accordance with the PRISMA 2020 guidelines [20]. The review protocol was prospectively registered with the PROSPERO database (registration number: CRD42023477692). The PRISMA 2020 checklist is provided in Supporting Table S1.

### 2.1. Eligibility Criteria

The eligibility criteria were structured using the PICOS framework (Population, Intervention or Independent variables, Comparators, Outcomes, and Study design). The study population included older adults (≥ 60 years) of both sexes. The independent variables were spontaneous PA or SB objectively measured using wearable devices (activity monitors or accelerometers). The comparators, when applicable, included groups with and without frailty. In studies without defined group comparisons, the associations between PA or SB and frailty scores were considered. Therefore, the outcome was the correlation between PA or SB and FS. Finally, the included studies required a cross‐sectional or cohort study design. Experimental studies that implemented specific exercise programs and those published in languages other than English were excluded.

### 2.2. Search Strategy

The sources of information were obtained by searching the PubMed/Medline, Web of Science, and Cochrane Library databases using the following search equation based on the PICOS criteria: [P/C Aged OR Home Nursing OR Institutionalization AND I=Wearable electronic devices OR Exercise OR Sedentary behavior AND O=Frailty AND S=Cross‐Sectional Studies OR Cohort Studies]. The equation for the full search can be found in the Supporting Information (Table S2). All articles published until August 30, 2025, were considered. Additionally, a backward search of the bibliographies of recent studies and reviews was conducted to include other relevant articles.

### 2.3. Study Selection

The study selection was a three‐stage process, with the identified citations independently evaluated for inclusion by two reviewers. The first stage was the evaluation of titles using the systematic equation described above. In the first stage, the article was included through title screening to identify frail participants. In the second stage, we reviewed the abstracts of all articles that met the search criteria. In the third stage, full‐text articles that met the criteria were retrieved and read independently by both reviewers and assessed for inclusion in the study. Disagreements were resolved by consensus between the two reviewers and a third reviewer if a consensus could not be reached. The reference sections of the relevant articles were examined using the collaborative web application Rayyan [21].

### 2.4. Data Extraction

The following information was extracted from the selected articles: study source (authors, journal, and year of publication), study design, number of participants, participant characteristics (age, sex, body mass, and BMI), and level of PA and SB time (only objectively measured using PA and SB using wearable devices). Simultaneously, the main outcome of interest was the FS index of older participants. In addition, data that were not available from tables or the results section and the respective authors of the studies were contacted by email, with one reminder after 2 weeks if they did not respond to the first email.

### 2.5. Risk of Bias and Study Quality Assessment

The study quality and risk were assessed using a 14‐item scale following the guidelines of the National Institutes of Health (NIH) Quality Assessment Tool for Observational Cohort and Cross‐Sectional Studies, which detects potential flaws in the study’s method or implementation [22].

To align the risk‐of‐bias judgments with the quantitative synthesis, the ROBINS‐I tool for nonrandomized studies was applied exclusively to the subset of studies that contributed effect sizes to the meta‐analyses (i.e., those with convertible outcomes). Since all included studies were observational, we used ROBINS‐I; RoB 2 is not suitable for nonrandomized evidence. This tool evaluates bias across seven domains: confounding, selection of participants, measurement of the exposure, measurement of outcomes, missing data, selection of the reported results, and leading to an overall risk‐of‐bias judgment. Confounding and selection bias were assessed based on control of prognostic variables and inclusion mechanisms. Exposure and outcome measurement domains examined validity, reliability, and blinding. Missing data and selective reporting were also evaluated. Overall risk of bias corresponded to the highest risk identified across domains, according to ROBINS‐I guidance [23].

### 2.6. Statistical Analysis

When identifying trials that measured the mean differences (MDs) of PA and SB between different frailty levels, following the Cochrane Handbook guidelines [24], MD was converted into correlation coefficients (*r*) ranging from −1 (*perfect inverse correlation*) to 1 (*perfect positive correlation*). This enabled a meta‐analysis of the isolated and pooled correlation coefficients between the directly measured PA and SB with FS. First, MDs were standardized using the pooled standard deviation: SD_pooled = √[(SD_1_^2^ + SD_2_^2^)/2], in order to obtain Cohen’s *d*. This value was then converted into a correlation coefficient using the formula r = *d*/√(*d* ^2^ + 4) [25].

The correlation coefficients between older participants’ PA, SB, and FS were summarized in forest plots with individual and pooled outcomes. For this purpose, a correlation analysis of the obtained correlation coefficients between the associations between objectively measured PA levels, SB time, and older frailty indices was performed.

For the main meta‐analysis outputs, the Cochran Q test was used to measure heterogeneity and *I* ^2^ statistics to measure inconsistency between individual study outputs, ranging from 0 (*any inconsistency*) to 100% (*maximal inconsistency*) [26]. Tau‐squared (*τ* ^2^) was measured to verify the between‐study variance. After verifying methodological heterogeneity between the included studies, random effects correlation meta‐analysis was applied to measure the 95% confidence interval (CI) for the correlation of the spontaneous and objectively measured PA and their FS [26].

Funnel plot analysis was performed to measure the publication bias. Subgroup analyses were performed to explore heterogeneity according to characteristics at the level of the studies, with the studies being stratified by device placement. Random‐effects meta‐analyses of correlation coefficients were conducted using the metacor function in R, a statistical software program. Fisher’s z transformation stabilized the correlations, REML estimation provided unbiased variance estimates, and Hartung–Knapp adjustment accounted for uncertainty in small samples [27]. Subgroup differences were formally tested, and the results displayed as forest plots showing the pooled subgroup estimates [28]. To verify sensitivity, the influence of individual studies on the pooled correlation meta‐analysis effects was determined. A sensitivity analysis was conducted to evaluate the study interaction effects on the correlation meta‐analysis’s primary results using the leave‐one‐out method. After removing each study, the meta‐analysis allowed judgments about the reliability of correlation meta‐analyses [29]. As reliability criteria, the *I* ^2^ scores for between‐study heterogeneity were considered, adopting cutoffs of 0%–24% = low, 25%–49% = moderate, and 50%–70% = high heterogeneity across studies, as suggested by Higgins et al. [30].

All analyses were performed in R (Statistical Programming Language, Version 4.3.1 [26]).

## 3. Results

### 3.1. Study Selection

**FIGURE 1:**
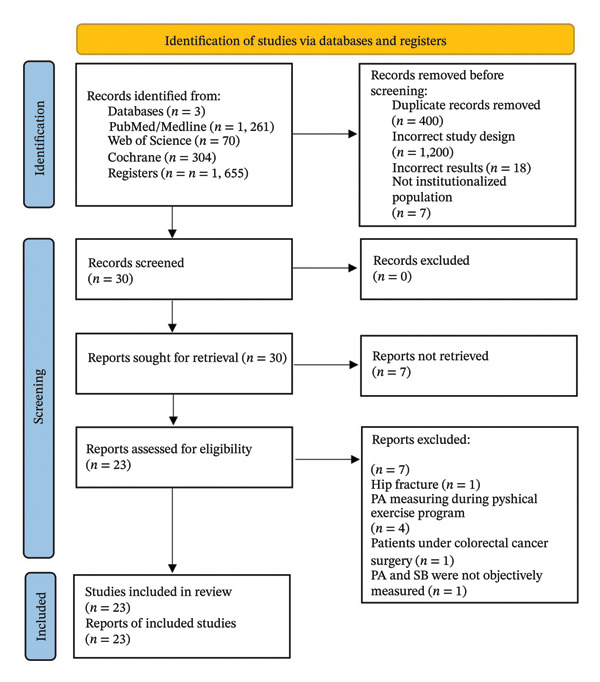
PRISMA flowchart of the systematic literature review.

Through a search of the PubMed/Medline, Web of Science, and Cochrane Library databases, 1655 records were identified. After eliminating 400 duplicate entries, 1225 records were excluded following an evaluation of their titles and abstracts for not meeting the eligibility criteria. The 30 remaining studies were assessed by reviewing the full text. Seven studies were excluded as they did not meet the inclusion criteria. Ultimately, 23 studies were included in this study (Figure 1).

### 3.2. Study Characteristics

In total, there were 10,244 participants in the 23 included studies, with a mean age ranging between 60 and 93 years. All studies had > 25 participants, with a total sample size ranging from 27 [31] to 2317 participants [15]. The subjects evaluated were older adults, and the follow‐up period for PA and SB measurements varied from 1 to 21 days (approximately 3 weeks). The independent variable was objective measurement of PA (*n* = 20) or SB (*n* = 16) using wearable devices. The methods used to assess FS, PA, and SB are shown in Table 1. All the included studies were cross‐sectional, with the exception of [32].

**TABLE 1:** Summary of results of objectively measured PA, SB, and frailty syndrome in older adults.

Author. year	Participants	Variables	Results: PA	Results: SB
Bastone et al. 2015	Primary care older adults (*n* = 28, 53.8% female) Age: 66–86 years	FS: self‐reported questionnaire‐based Fried criteria PA (steps/day) and SB (min/day): Actigraph GT3X (wear on the waist, 7 consecutive days)	Nonfrail (*n* = 13): 8085.7 ± 4205.0 steps/day Frail (*n* = 13): 2822.6 ± 2271.5 steps/day MD = 5263.1 (61%) *p* > 0.0001	Nonfrail (*n* = 13): 1046.3 ± 145.5 min/day Frail (*n* = 13): 1194.8 ± 125.1 min/day *p* = 0.01

Buckinx et al. 2017	Nursing home residents (*n* = 27, 75% female) Age: 86.7 ± 7.8 years BMI: 24.7 ± 5.2 kg/m^2^	FS: Fried criteria PA (steps per day): pebble + tracker (wear on the shoe, 7 consecutive days)	Group < 1300 steps/day (*n* = 14): 2.2 ± 0.9 points Group ≥ 1300 steps/day (*n* = 13): 0.8 ± 0.9 points MD = 1.4 points (63%) *p* = 0.0005	Not reported

Chen et al. 2020	Community‐dwelling older adults (*n* = 819, 51.8% female) Age: 70.9 ± 3.1 years	FS: FRAIL scale (Japanese version) PA (steps/day) and SB (min/day): Active style Pro HJA‐350IT (wear on the waist, 4 consecutive days)	Robust (*n* = 493): 5652.9 ± 2803.3 steps/day Frail group (*n* = 98): SPD = 4451.7 ± 3057.0 steps/day MD = 1201.2 (21%) *p* < 0.001	Robust group (a: *n* = 493): 460.1 ± 113.0 min/day Prefrailty (b: *n* = 228): 450.7 ± 104.4 min/day Frailty (c: *n* = 98): SB = 455.3 ± 118.7 min/day MD^ab^ = 9.4 (2.04) MD^bc^ = 4.6 (1%) MD^ac^ = 4.8 (1%) *p* > 0.05

Din et al. 2020	Village resident older people (*n* = 59, 63% female) Age: 73.9 ± 11.2 years	FS: comprehensive geriatric assessment PA (steps/day): Axivity AX3 (5^th^ lumbar vertebra, 7 consecutive days)	Nonfrail (*n* = 36): 8409.18 ± 5916.35 steps/day Frail (*n* = 10): 1029 ± 1432.73 steps/day MD = 7380.18 (87.7%), *p* = 0.001	Not reported

Kikuchi et al. 2018	Community‐dwelling older adults (*n* = 511, 53% female) Age: 73.4 ± 5.6 years	FS: J‐CHS PA (min/day moderate to vigorous) and SB: (min/day): Active style Pro HJA‐750C (hip, 7 consecutive days)	Robust (*n* = 264): 58.6 ± 40.1 min/day Frail (*n* = 13):14.9 ± 21.1 min/day MD = 43.7 (33.2%) *p* < 0.0001	Robust (a: *n* = 264): 58.6 ± 40.1 min/day Prefrailty MPD (b: *n* = 234): SB = 186 ± 110 min/day Frailty: (c: *n* = 13), SB = 288.9 ± 158.7 ^c^ min/day MD^ab^ = 127 (68.2%) MD^bc^ = 102.9 (64.3%) MD^ac^ = 230.3 (79.7%) *p* < 0.0001^ab^

Kumar et al. 2020	Older adults being served in primary, secondary, and tertiary healthcare settings (*n* = 104, 80.1% female) Age: 73.4 ± 5.6 years BW: 71.6 ± 15.4 kg	FS: Fried criteria PA (steps per day): PAMSys (shirt with a pocket at the sternum, 2 consecutive days)	Nonfrailty (*n* = 40): 1868 ± 1736 steps/day Prefrail/frail (*n* = 54): 897 ± 1056 steps/day MD = 971 (51.4%) *p* < 0.001	Not reported

Mañas et al., 2018	Institutionalized and community‐dwelling older adults (*n* = 519, 54.9% female) Age: 78.8 ± 4.6 years BMI: 30.5 ± 4.7 kg/m^2^	FS: Frailty Trait Scale PA (min/day of moderate to vigorous activity) and SB (min/day)	Without comorbidity: *R* ^2^ = 0.507, *p* < 0.01 With comorbidity: *R* ^2^ = 0.456∗, *p* < 0.01	Without comorbidity: *R* ^2^ = 0.548, *p* > 0.05 With comorbidity: *R* ^2^ = 0.442, *p* < 0.05

Nagai et al. 2018	Community‐dwelling older adults (*n* = 886, 70% female) Age: 73.6 ± 7 years; BMI: 22.7 ± 3.08 kg/m^2^	FS: Fried criteria PA (min/day moderate to vigorous) and SB (min/day): Actiband (wrist, 14 consecutive days)	Robust (a: *n* = 359): 45 ± 31 min/day Prefrail (*n* = 477):41 ± 34 min/day Frail (*n* = 50), MVP *A* = 21 ± 37 min/day MD^ab^ = 4 (8.8%) MD^bc^ = 20 (44.4%) MD^ac^ = 24 (63.3%) *p* > 0.001^a,b^	Robust (a: *n* = 359): 488 ± 153 min/day Prefrail (*n* = 477): 608 ± 169 min/day Frail (*n* = 50): 683 ± 197 min/day MD^ab^ = 120 (19.7%) MD^bc^ = 75 (11%) MD^ac^ = 195 (28.5%) *p* < 0.001^a,b^

Pozo‐Cruz et al. 2017	Community‐dwelling older adults (*n* = 519, 54.9%) female) Age: 78.84 ± 4.55 years BMI: 30.54 ± 4.74 kg/m^2^	FS: Frailty Trait Scale SB (min/day): ActiGraph, ActiTrainer 3X (hip, 7 consecutive days)	Not reported	Frailty proportion = 39.84 ± 15.12%. *R* ^2^ = 0.23, *p* < 0.05

Schwenk et al. 2015	Older adults being served in primary, secondary, and tertiary healthcare settings (*n* = 125, 20% female) Age: 79 ± 8 years	FS: Fried criteria PA: maximum continuous steps per day PAMSys (shirt with a pocket at the sternum, 2 consecutive days)	Robust (a: *n* = 44): 352 ± 375 Prefrail (b: *n* = 60): 183 ± 220^b^ Frail (c: *n* = 21): 83 ± 67^c^ MD^ab^ = 169 (48%) MD^bc^ = 100 (28.4%) MD^ac^ = 269 (76.4%) *p = *0.01	Not reported

Song et al. 2015	Ambulatory older adults (*n* = 1333, 67.2% female) Ag: 67 ± 7.7 years	FS: low gait speed (< 0.6 m per second) or inability to rise from a chair without using one’s arms were used to diagnose frailty SB (h/day): ActiGraph GT1M (hip, 7 consecutive days)	Not reported	*p* = 0.033

Schmidle et al. 2023	Institutionalized and community‐dwelling older adults (*n* = 88, 55% female), Age: 76.3–85 years BMI: 25.8–27.3 kg/m^2^	FS: self‐reported questionnaire‐based Fried criteria PA: 95th percentile of cadence in steps per minute (Huawei Watch 2, hip, 21 consecutive days)	Correlation between FS and PA: *R* ^2^ = 0.25, *p* < 0.001	Not reported

Castaneda‐Gameros et al. 2017	Older migrant women from ethnically diverse backgrounds (*n* = 60, 100% female) Age = 70.8 ± 8.1 years	FS: self‐reported questionnaire‐based Fried criteria PA (min/day) and SB (min/day): Actigraph GT3X (hip, 7 consecutive days)	Nonfrail (*n* = 23): 18.4 ± 19.9 min/day Frail (*n* = 10): 3.4 ± 4.5 min/day MD = 15 (19%) *p* < 0.01	Nonfrail (*n* = 23): 523.7 ± 85.7 min/day Frail (*n* = 10): 576.7 ± 7 min/day MD = 53 (9.1%) *p* = 0.48

Higueras‐Fresnillo et al. 2020	High‐functioning commun0.23‐dwelling older adults (*n* = 436, 65.8% female) Age: 71.6 ± 5.28 years; BW: 71.07 ± 12.79 kg	FS: self‐reported questionnaire‐based Fried criteria PA (min/day) and SB (min/day). IDEEA (sternum, 1 day)	*p* < 0.01	*p* > 0.05

Kehler et al. 2018	Community‐dwelling older adults (*n* = 2317, 49.3% female) Age: 67.4 ± 0.32 years; BW = 80.0 ± 0.5 kg	FS: accumulation of deficits model FI PA (min/week) and SB (min/day): Actigraph 7164 (hip, 7 consecutive days)	*p* > 0.0002	*R* ^2^ = 0.26, *p* = 0.0007

Razjouyan et al. 2018	Ambulatory older adults (*n* = 111, 79% female) Age: 75 ± 10 years	FS: self‐reported questionnaire‐based Fried criteria PA (steps/month) and SB (h/day): PAMSys (sternum, 2 consecutive days)	Prefrail (*n* = 78): 47.7 ± 30.7 Frail (*n* = 33): 11.2 ± 14.6 MD = 36.5 (23.4%) *p* > 0.04	Prefrail (*n* = 78): 11.7 ± 3.2 h/day Frail (*n* = 33): 13.2 ± 4.2 h/day MD = 1.5 (11.3%) *p* = 0.02, *d* = 0.40.

Ting Li et al. 2022	Ambulatory older adults (*n* = 1099, 100% female), Age: 63–67 years BMI: 25 (23.2–27.4)	FS: self‐reported questionnaire‐based Fried criteria SB (h/day): Actigraph Wgt3x‐bt (hip, 7 consecutive days)	Not reported	Nonfrail (a: *n* = 459), 520.5 [465.6 to 581.0] Prefrail (b: *n* = 578), 531.7 [477.0 to 602.7] Frail (c: *n* = 62), 584.8 [485.0 to 702.9] MD^ab^ = 11.2 (2.1%) MD^bc^ = 53.1 (9.1%) MD^ac^ = 64.3 (11%) *p* < 0.05

Theou et a. 2012	Ambulatory older adults (*n* = 50, 100% female) Age: 63–90 years	FS: accumulation of deficits PA (min/day): Polar WearLink 31 (chest with elastic belt, 2 days)	Low frailty (a: *n* = 16): 3599 ± 1781 min/day Intermediate frailty (b: *n* = 17): 1773 ± 1048 min/day High frailty (c: *n* = 17), 873 ± 809 min/day MD^ab^ = 1826 (50.7%) MD^bc^ = 900 (25%) MD^ac^ = 2726 (75.7%) *p* > 0.05^a,b^	Not reported

Wanigatunga et al. 2022	Healthy and fragile older adults (*n* = 567, 44% female) Age: 76.5 ± 5.4 years; BMI: 30.6 ± 6.0 kg/m^2^	FS: self‐reported questionnaire‐based on Fried criteria PA and SB (h/day): Actigraph GT9X, wrist, 7 consecutive days)	OR = 1.07 (1.03–1.10), *p* < 0.01.	*p* < 0.05.

Yuki et al. 2019	Care facility resident older adult (*n* = 401, 44.4% female) Age: 71.1 ± 4.3 years	FS: self‐reported questionnaire based on Fried criteria PA (steps/day): Lifecorder (not recorded, 7 consecutive days)	*p* < 0.01	Not reported

Ziller et al. 2020	Care facility resident older adult (*n* = 47, 66% female) Age: 74 ± 6 years, BMI: 28.6 ± 4.9 kg/m^2^	FS: self‐reported questionnaire‐based Fried criteria PA (min/week) and SB (h/day): ActiGraph Wgt3xbt (right hip, 7 consecutive days)	Nonfrail (a: *n* = 23), 252 ± 158 min/week Prefrail (b: *n* = 15), 129 ± 210 min/week Frail (c: *n* = 9), 26 ± 14 min/week MD^ab^ = 123 (48.8%) MD^bc^ = 103 (40.8%) MD^ac^ = 226 (89.6%) *p* = 0.003	Nonfrail (a: *n* = 23), 9.7 ± 1.8 (69.9% ± 7.9%) h/day Prefrail (*n* = 15), 9.9 ± 3.3, (74.4% ± 9.4%) h/day Frail (*n* = 9), 10.3 ± 2.8, (78.6% ± 6.0%) h/day MD^ab^ = 0.2 (2.02%) MD^bc^ = 0.4 (4%) MD^ac^ = 0.6 (6%) *p* = 0.028

Camerlingo et al. 2023	Community‐dwelling older adults (*n* = 50, 64% female) Age: Nonfrail (*n* = 21): 71.10 ± 3.59 years Prefrail (*n* = 23): 73.74 ± 5.52 years Frail (*n* = 6): 70.70 ± 6.53 years	FS: Fried frailty score PA: GENEActiv wrist‐worn accelerometer (2 weeks at‐home) SB: GENEActiv wrist‐worn accelerometer (wrist and lumbar, 2 weeks)	Nonfrail (a: *n* = 21), 1.88 ± 0.96 h/day Prefrail + frail (b: *n* = 28), 1.15 ± 0.68 h/day MD^ab^ = 0.73 h/day (39%) *p* = 0.01	Nonfrail (a: *n* = 21), 11.4 ± 1.61 h/day Prefrail + frail (b: *n* = 28), 12.3 ± 1.88 h/day MD^ab^ = 0.9 h/day (7.9%) *p* = 0.127

Park et al. 2022	Community‐dwelling older adults (*n* = 88) Nonfrail (*n* = 47, 51.1% male): 70.72 ± 3.64 years, BMI: 23.82 ± 2.50 kg/m^2^ Frail (*n* = 11, 9.1% male): 77.73 ± 6.17 years, BMI: 27.16 ± 4.23 kg/m^2^	FS: Fried criteria PA (duration by intensity): Fitbit Alta HR (wrist, 7 days) SB: Fitbit Alta HR	Nonfrail (a: *n* = 47), 60.47 ± 40.78 min/day Frail (b: *n* = 11), 18.76 ± 15.85 min/day MD^ab^ = 41.71 min/day (69%) *p* = 0.01	Nonfrail (a: *n* = 47), 655.06 ± 136.90 min/day Frail (b: *n* = 11), 728.50 ± 111.74 min/day MD^ab^ = 73.44 min/day (11%) *p* = 0.19

*Note: β*: regression coefficients; EMG: electromyography; F: F‐statistics; GEE: generalized estimation equation models; IQR: interquartile range; J‐CHS: Cardiovascular Health Study criteria for Japanese older adults; *R* ^2^: coefficient of determination; X^2^: chi‐square test.

Abbreviations: BMI, body mass index; BW, body weight; CI, confidence interval; FS, frailty status; GPS, global positioning system; HR, hazard ratio; IDEEA, Intelligent Device for Energy expenditure and Activity; LPA, low‐intensity PA; OR, odds ratio; PA, physical activity; SB, sedentary behavior.

### 3.3. Quality and Risk of Bias

All studies presented a minimal risk of bias, and their quality was considered good according to the NIH Quality Assessment Tool (score > 11 points). In all 23 included studies, the outcome assessors were not blinded to the participants′ exposure status. Only in the study by Yuki et al. [32] was there a 20% loss to follow‐up after the start of the study. Higueras‐Fresnillo et al. [33] did not report the inclusion and exclusion criteria. Additionally, 11 studies did not justify the sample size [15, 32–41] (Table 2).

**TABLE 2:** Methodological quality and risk of bias in individual studies.

Study	1	2	3	4	5	6	7	8	9	10	11	12	13	14
Buckinx et al. 2017														
Chen et al. 2020														
Kikuchi et al. 2018														
Kumar et al. 2020														
Nagai et al. 2018														
Schwenk et al. 2015														
Mañas et al. 2018														
Schmidle et al. 2023														
Carvalho Bastone et al. 2015														
Castaneda‐Gameros et al. 2017														
Din et al. 2020														
Higueras‐Fresnillo et al. 2020														
Kehler et al. 2018														
Razjouyan et al. 2018														
Pozo‐Cruz et al. 2017														
Song et al. 2015														
Theou et al. 2011														
Ting Li et al. 2022														
Wanigatunga et al. 2022														
Yuki et al. 2019														
Ziller et al. 2020														

*Note:* Item 1: research question. Items 2 and 3: study population. Item 4: groups recruited from the same population and uniform eligibility criteria. Item 5: sample size justification. Item 6: exposure assessed prior to outcome measurement. Item 7: sufficient timeframe to see an effect. Item 8: different levels of the exposure of interest. Item 9: exposure measures and assessment. Item 10: repeated exposure assessment. Item 11: outcome measures. Item 12: blinding of outcome assessors. Item 13: follow‐up rate. Item 14: statistical analyses. Green: yes. Red: no. Level of quality considered good: > 11 points.

**FIGURE 2:**
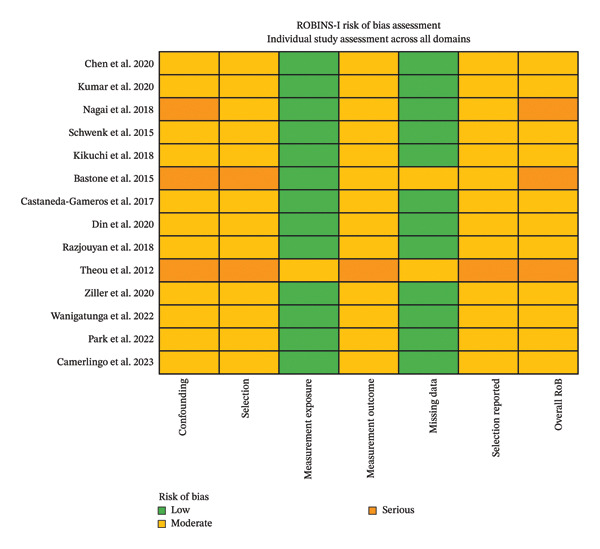
Individual study assessment across all domains of the ROBINS tool.

**FIGURE 3:**
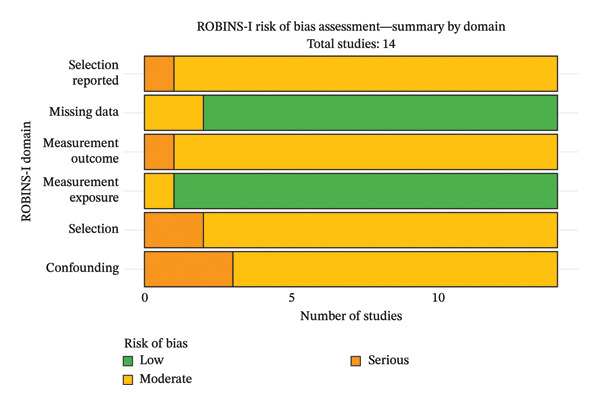
Summary by domain of the ROBINS tool.

**FIGURE 4:**
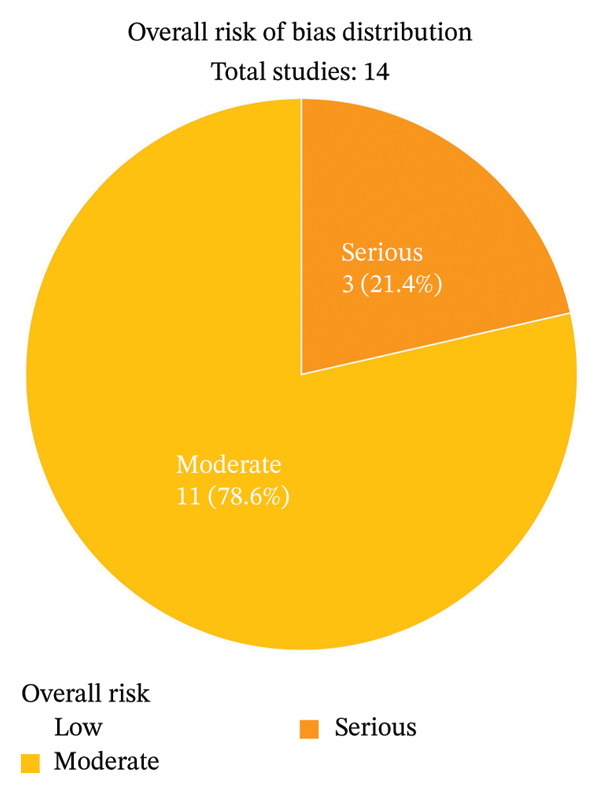
Percentage of studies by level of risk of bias according to the ROBINS tool.

Analyses using the ROBINS‐I tool, applied exclusively to the meta‐analyzed studies, showed that only the studies Bastone et al. (2015), Nagai et al. (2018), and Theou et al. (2012) [39, 42, 43] reported components of serious risk of bias. The Nagai et al. study [43] had a serious risk of bias for the “controlling for possible confounding factors” domain, the Bastone et al. study [42] had a serious risk of bias for the “controlling for possible confounding factors” and “participant selection” domains, and the Theou et al. study [39] had a serious risk of bias for the “controlling for possible confounding factors,” “participant selection,” “measurement outcome,” and “selection reported” domains (Figures 2 and 3). Overall, 3/14 studies (21%) were ranked with a serious risk of bias and 11/14 (79%) with a moderate risk of bias (Figure 4).

### 3.4. Results of Individual Studies

#### 3.4.1. Correlation Between Objectively Measured PA and FS

All studies (*n* = 20) reported statistically significant inverse effects of PA and FS in older adults. Effect sizes varied widely across studies: ORs ranged from 0.999 to 1.80, *β* coefficients from −0.110 to 6.20, and MDs from 21% to 89.6%. For detailed estimates and CI from each study, see Table 1.

#### 3.4.2. Correlation Between Objectively Measured SB and FS

Among the 16 studies that evaluated SB, 13 studies presented significant and positive associations between SB and older FS [15, 16, 35–38, 40–44]. Exceptions include the studies of Chen et al. [19], Castaneda‐Gameros et al. [45], and Higueras‐Fresnillo et al. [33].

Effect sizes across studies varied considerably. ORs ranged from 1.025 to 1.14, *β* coefficients from −0.80 to 2.556, hazard ratios (HRs) from 0.95 to 1.36, and MDs from 1% to 79.7% change. All reported effects were statistically significant at *p* < 0.05 or better. For full estimates, CI, and *R* ^2^ values per study, see Table 1.

### 3.5. Meta‐Analysis Results

Regarding the correlation meta‐analysis results of PA’s correlation with the older adults’ FS, of the 20 included studies (*n* = 7293), only 14 (*n* = 2100 participants, 29%) were meta‐analyzed because the other 6 did not present convertible outputs [24].

**FIGURE 5:**
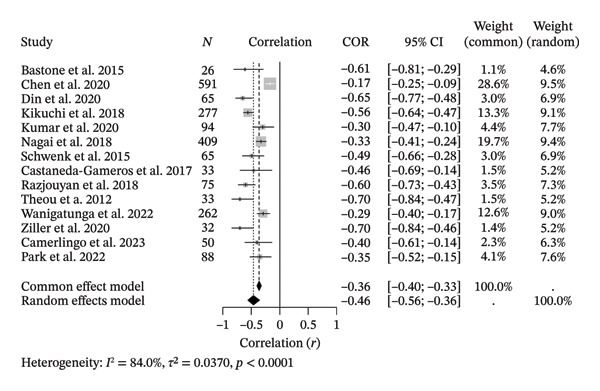
Correlation meta‐analysis between spontaneously measured physical activity and frailty syndrome in older adults.

**FIGURE 6:**
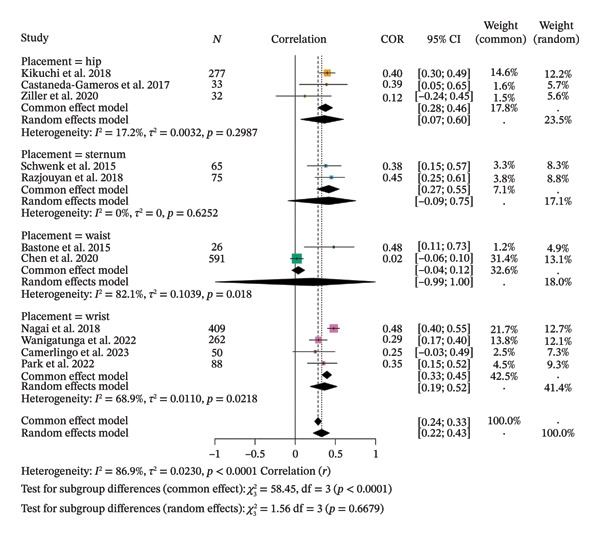
Subgroup analysis of the correlation meta‐analysis between spontaneously measured physical activity and frailty syndrome in older adults.

The 14 included studies presented significant inverse correlations between spontaneously measured PA and FS (*r* = −0.48, CI = [−0.5585; −0.3560], *Z* = −8.39, *p* < 0.0001) [18, 19, 34, 37, 39–48]. These correlation coefficients ranged from *r* = −0.17 to *r* = −0.70. These results corresponded to a random‐effects correlation meta‐analysis since the Cochran’s Q test indicated high heterogeneity (*I* ^2^ = 86%, CI = 84.0% [74.5%; 89.9%]; with the corresponding standard deviation of the reported correlation coefficients during the random effects model, *H* = 2.50, CI = [1.98; 3.15], and the *τ* ^2^ test reached a score of 0.0441, CI = [0.0132; 0.1152]; tau = 0.1923, CI = [0.1149; 0.3394], indicating statistically significant between‐study variance, confirmed by Cochran’s *Q* test, *Q* = 81.04, df = 13, *p* < 0.0001 (Figure 5). Subgroup correlations were significant for lumbar vertebrae (*r* = −0.6500), chest (*r* = −0.7000), hip (*r* = −0.5656), sternum (*r* = −0.4678), and wrist (*r* = −0.3236), while waist showed a nonsignificant correlation (*r* = −0.3812). Between‐group differences were significant (*Q* = 45.22, df = 5, *p* < 0.0001; Figure 6).

Regarding SB, also due to missing convertible data, from the 16 included studies (*n* = 9390), 11 studies were meta‐analyzed (*n* = 1908, 20%).

The pooled correlation meta‐analysis identified a significant and positive correlation between objectively measured SB and FS (*r* = 0.334, CI = [0.2233; 0.4259], *Z* = 6.67, *p* < 0.0001).

**FIGURE 7:**
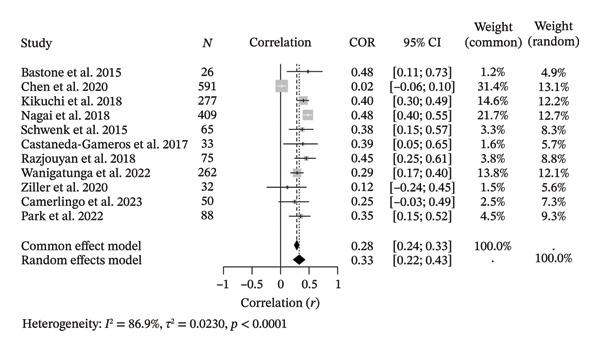
Correlation meta‐analysis between spontaneously measured sedentary behavior and frailty syndrome in older adults.

Of the 11 included studies, eight presented significant isolated correlations [18, 37, 40, 42–45, 47], whereas Chen et al. [19] (*r* = 0.0200, CI = [−0.0607; 0.1005]), Park et al. (2022) (*r = *0.3500, CI = [0.1517; 0.5212], and Ziller et al. (2020) [41] (*r* = 0.1200, CI = [−0.2387; 0.4499]) did not. Correlation coefficients ranged from *r* = 0.02 to *r* = 0.48. These results also corresponded to a random‐effects meta‐analysis, as indicated by the high heterogeneity Cochran Q test percentage scores, *I* ^2^ = 86.9%, CI = [78.4%; 92.0%], with the corresponding standard deviation of the reported correlation coefficients during the random effects model, *H* = 2.76, CI = [2.15; 3.54], and the *τ* ^2^ test reached a score of 0.0230, CI = [0.0065; 0.0686]; tau = 0.1516, CI = [0.0808; 0.2618], indicating statistically significant between‐study variance, confirmed by Cochran’s *Q* test, *Q* = 76.35, df = 10, *p* < 0.0001 (Figure 7).

**FIGURE 8:**
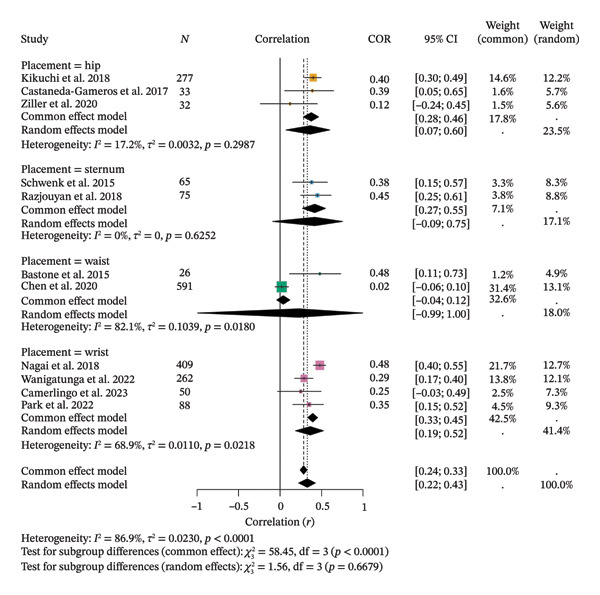
Subgroup analysis of the correlation meta‐analysis between spontaneously measured sedentary behavior and frailty syndrome in older adults.

Regarding subgroup analysis, the 11 included studies showed positive correlations between SB and FS by device placement, ranging from *r* = 0.0389 to *r* = 0.4182. Random‐effects meta‐analysis yielded a pooled *r* = 0.3284 (95% CI [0.2233; 0.4259], *z* = 6.67, *p* < 0.0001) with high heterogeneity (*τ* ^2^ = 0.0230, *τ* = 0.1516, *I* ^2^ = 86.9%, *H* = 2.76; *Q* = 76.35, df = 10, *p* < 0.0001). Subgroup correlations were significant for hip (*r* = 0.3642) and wrist (*r* = 0.3653), while sternum (*r* = 0.4182) and waist (*r* = 0.2260) were nonsignificant. Between‐group differences were not significant (*Q* = 1.56, df = 3, *p* = 0.6679) (Figure 8).

### 3.6. Publication Bias Assessment

**FIGURE 9:**
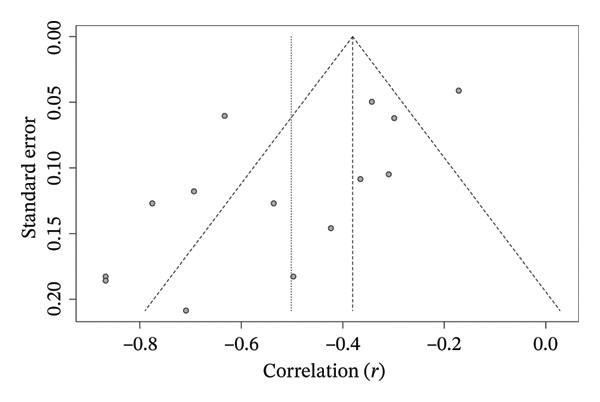
Funnel plot of correlation meta‐analysis between spontaneously measured physical activity and frailty syndrome in older adults. The *x*‐axis shows the correlation coefficient, and the *y-*axis shows the standard error between the studies.

Funnel plot analysis about the PA’s correlation with the older adults’ FS revealed asymmetry in the distribution of standard errors across studies with varying sample sizes, explaining the heterogeneity between the correlation coefficients [*t* = −3.09, df = 12, *p* = 0.0094, bias estimate: −3.2709 (SE = 1.0597)]. These asymmetries showed that studies with smaller sample sizes had the largest effect sizes (Figure 9).

**FIGURE 10:**
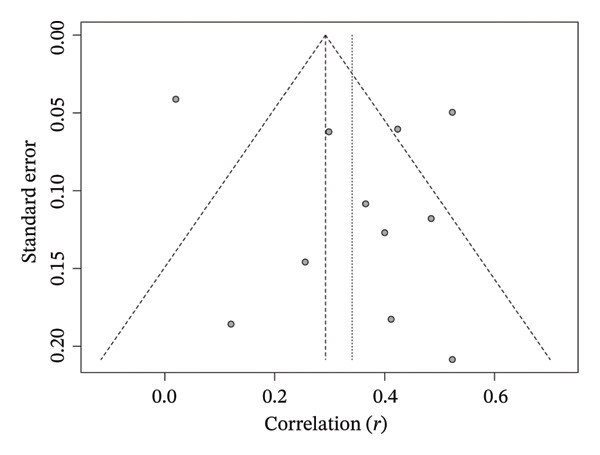
Funnel plot of correlation meta‐analysis between spontaneously sedentary behavior and frailty syndrome in older adults. The *x*‐axis shows the correlation coefficient, and the *y*‐axis shows the standard error between the studies.

Funnel plot analysis of SB’s correlation with older adults’ FS revealed no significant asymmetry in the distribution of standard errors across studies with varying sample sizes [*t* = 0.89, df = 9, *p* = 0.396, bias estimate = 1.5304 (SE = 1.7155)]. These results indicate that smaller studies did not systematically report larger effect sizes (Figure 10).

### 3.7. Sensitivity Analysis

**FIGURE 11:**
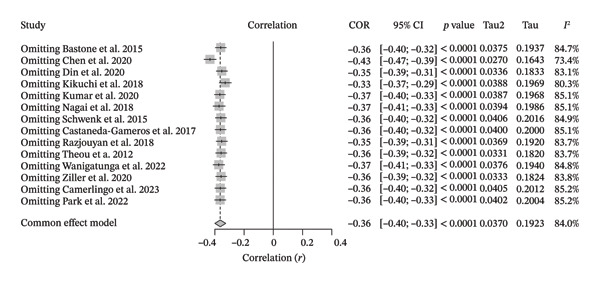
Sensitivity analysis for the correlation meta‐analysis between objectively measured physical activity and frailty syndrome.

The sensitivity analysis showed that, even after the leave‐one‐out application, the *I* ^2^ scores remained high for the correlation analysis between PA and FS (pooled *I* ^2^ = 84%, *I* ^2^ range = [73.4–85.2]). The pooled *r* ranged from −0.33 to −0.43 (Figure 11). Only one study [19] caused absolute changes in the *I* ^2^ values, decreasing them from 85.2% to 73.4% and increasing the correlation from −0.33 to −0.43. Nevertheless, these changes are not considered significant since the *I* ^2^ values remained in the “high heterogeneity” classification across studies, as suggested by Higgins et al. [30].

**FIGURE 12:**
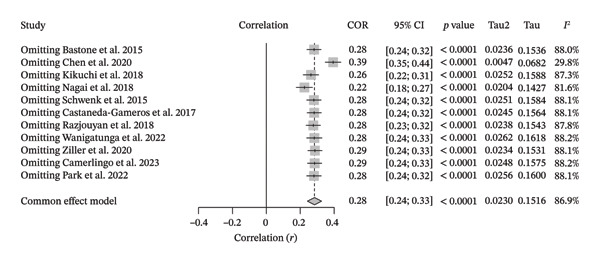
Sensitivity analysis for the correlation meta‐analysis between objectively measured sedentary behavior and frailty syndrome.

For the association between SB and FS, the sensitivity analysis showed a significant reduction in heterogeneity after the leave‐one‐out application, with the pooled *I* ^2^ decreasing from 88.1% to 29.8%. The pooled correlation coefficient and its range increased from 0.22 to 0.39. Specifically, for SB, this reduction was driven by the removal of the Chen et al. study [19] (Figure 12).

## 4. Discussion

This systematic review and meta‐analysis aimed to analyze the correlation between spontaneous PA and SB objectively measured using wearable devices and FS in older adults. Our main finding is that spontaneous PA and FS have a significant inverse correlation, whereas SB and FS have a significant positive correlation, thus supporting our hypothesis.

Our results demonstrate that higher activity levels are significantly associated with a lower likelihood of frailty. Similarly, Tolley et al. [17] verified that PA was significantly inversely related to FS in older adults. Reduced daily PA could harm older people’s health by aggravating FS and its progression [19, 31, 49]. Breen et al. [50] found that 2 weeks of reduced PA was sufficient to reduce muscle protein synthesis by 26%, accompanied by significant reductions in leg free fat mass concentrations. Similarly, McGlory et al. [51] found that 2 weeks of reduced activity caused a 12% reduction in muscle protein synthesis and increased insulin resistance and inflammation markers, and muscle protein synthesis is an essential physiological process to maintain proteostasis and good muscle function [52]. Older people are more likely to have deregulated proteostasis, which reduces their muscle size and function, that is, catabolic processes, and worsens muscle metabolism [53]. These alterations put the less active older adults at higher risk of sarcopenia and FS [54]. In a prospective and observational study, Mora et al. [55] verified that increased PA levels during an 11‐year follow‐up period significantly reduced the participants’ cardiovascular risk due to a reduction in inflammatory markers, blood pressure, blood lipid levels, body mass index, and diabetes. Controlling cardiovascular factors through a physically active lifestyle is also a determining factor in reducing FS risk in older adults [4].

Sedentarism is a behavioral condition characterized by decreased or lying positions that consequently lead to musculoskeletal system disuse, implying negative adaptations for overall health, such as muscle atrophy, loss of mobility, and decreased muscle endurance [56]; bone loss that could lead to osteopenia and osteoporosis [57]; and metabolic issues such as weight gain, increased overall body fat mass and visceral fat mass, and insulin resistance onset [58], which increase cardiovascular disease risk [59]. In addition, high SB time is also linked to reduced cognitive function, which limits older adults from independently performing their ADLs [60]. Furthermore, Middleton et al. [61] performed a cohort study with 890,000 patients aged 75 years and over and found a positive association between SB and an increased risk of falls, fractures, and early mortality. Additionally, two systematic reviews [15, 16] found a significant number of studies reporting a significant and direct association between SB and FS in older adults. Therefore, our results showed that SB was associated with FS, strengthening the body of evidence on this association in older adults.

This evidence provides crucial practical applications for healthcare providers, including physiotherapists, specialists in PA and sports sciences, nurses, and doctors. In this population, strategies should be implemented to increase daily PA, such as promoting higher daily step counts [62], as a key target for interventions aimed at preventing frailty in older adults. This recommendation is in line with World Health Organization Guidelines on Physical Activity and Sedentary Behavior, which encourage that older adults integrate PA across all domains of daily living [63]. In this context, wearable devices can serve as standardized and objective tools to monitor and manage frailty risk by providing proxy indicators of frailty status. This approach enables decentralized care and significantly reduces the need for frequent travel to clinical sites, which are often burdensome and exhausting for older individuals. For accurate clinical interpretation, it is essential to consider activity data alongside device wear time and sleep patterns, as reductions in sedentary time may reflect lower device compliance or increased sleep rather than true behavioral changes. Recent evidence shows that these recommendations can be implemented through healthcare programs based on artificial intelligence and the internet of things, as demonstrated by Kim & Kim [64]. In their study, a 6‐month intervention using wearable devices, weekly health monitoring, and personalized feedback significantly increased walking frequency and other PA metrics in older adults.

This study has some important strengths. This first meta‐analytic approach, which included only objectively measured PA and SB through wearable devices, was confirmed and is reliable in reporting these metrics measured in older people [11]. Secondly, despite the funnel plot analysis indicating publication bias for PA, only the study by Chen et al. [19] considerably changed the correlation meta‐analysis for PA and FS [*r* = 0.36 to 0.43] and for SB and FS [*r* = 0.28 to 0.39]. Despite this influence from individual studies, both correlations remained in the moderate effect classification, with the final inferences of this meta‐analysis practically unchanged. Moreover, after leave‐one‐out sensitivity analysis, the method did not indicate any influence of individual studies for either the PA or SB meta‐analyses.

Nonetheless, our study has some limitations that must be noted. First, the studies included in our meta‐analysis used different frailty indices (e.g., Fried criteria, FRAIL scale, and Frailty Trait Scale). While all measure the overarching concept of frailty, differences in their specific criteria and scoring could introduce heterogeneity in the outcome measures, potentially affecting the consistency of the pooled estimate. Similarly, the included studies used different definitions of PA and SB, reflecting various components of activity such as total volume, intensity, or gait characteristics. To achieve a higher level of evidence, we included these studies despite the differences in definitions, combining their measures in the meta‐analysis. Moreover, PA and SB were measured using different devices, which could reduce the reliability of the effect‐size conversion, and the devices were worn in different body locations and in different ranges of days throughout the studies, which can affect the validity of the results [65]. Additionally, the amount of data included in the meta‐analysis was limited because some authors did not provide measures of central tendency or raw data that should help increase the reliability and consistency of the meta‐analysis output. Specifically, the meta‐analysis of the correlation between PA and FS included 14 studies. While this is a relatively small number, we selected a random‐effects model because it is the most appropriate approach for heterogeneous data, as it accounts for both within‐study and between‐study variance and provides a more conservative and generalizable estimate [66]. Although meta‐regression with moderators such as device, placement, frailty instrument, setting, and wear time was considered, the limited number of studies precluded reliable modeling. In line with Cochrane Handbook recommendations (minimum of 10 studies per covariate), we opted for subgroup and sensitivity analyses to ensure more stable and reliable estimates [24]. Additionally, the higher heterogeneity reported for both PA and SB analyses may be explained by the substantial variation in outcome assessment methods across studies, which can directly influence the degree of variability in the reported results of each study [67].

Furthermore, the included studies were performed on different populations, which can be considered a bias factor. Moreover, most of the included studies were cross‐sectional (*n* = 22), thus limiting our inferences regarding causal effects. Finally, all included studies presented a nonblinded process for the outcome assessors, which in turn can significantly bias and overestimate the meta‐analysis estimations by an average of 29% [68].

Finally, this systematic review has also certain limitations pertaining to the literature search. Although we searched three major databases (PubMed, Web of Science, and Cochrane Library), it is possible that some relevant studies were not captured, as additional databases were not consulted. Furthermore, the restriction to publications in English may introduce a language bias, potentially excluding relevant evidence published in other languages. However, this risk is likely mitigated by the fact that the most influential research in this field is predominantly published in English.

Future systematic reviews and meta‐analyses can consider using the most possible standardized data, including a higher number of studies. Therefore, we suggest performing a dose–response meta‐analysis between objectively measured PA and SB to verify the minimal dose associated with PA and SB for frailty in older adults. Furthermore, future investigations can also consider the reverse causality, where subjects with advanced frailty levels may experience increased difficulties in increasing their PA levels [69]. To avoid inverse causality, new research can address methodological limitations, such as assessor blinding, as well as the preferential use of accurate evaluation methods and participant selection and allocation. Therefore, the application of more robust analytic methods, such as causality decomposition, can help infer the most relevant factors to the target outcome [70]. Finally, the production of longitudinal designs will increase the body of evidence regarding the effects of PA and SB in older adults’ FS and will contribute to future meta‐analyses.

## 5. Conclusion

In conclusion, this systematic review and meta‐analysis showed that spontaneous PA is inversely correlated with FS and that SB is positively correlated with FS. These findings provide essential evidence for healthcare professionals—including physiotherapists, specialists in PA and sports sciences, nurses, and physicians—to consider increasing PA as a key target for interventions aimed at preventing and managing frailty in older adults.

## Funding

This study was supported by the Emerging Doctors Project 2024 UAM (SI4/PJI/2024‐00160) to Helios Pareja Galeano.

## Ethics Statement

No ethical approval was required, as this study relies solely on published data.

## Conflicts of Interest

The authors declare no conflicts of interest.

## Supporting Information

Additional supporting information can be found online in the Supporting Information section.

## Supporting information


**Supporting Information** The supporting information includes the PRISMA 2020 reporting checklist (Table S1) and the complete search strategies used across PUBMED/MEDLINE, Web of Science, and Cochrane databases (Table S2).

## Data Availability

The data presented in this study are available on request from the corresponding author.
