# Supplementary material for: Correlation Between Objectively Measured Spontaneous Physical Activity and Sedentary Behavior With Frailty Syndrome in Older Adults: A Systematic Review and Meta‐Analysis of Observational Studies
Source: J Aging Res. 2026 May 31;2026:6085101. doi: 10.1155/jare/6085101 (PMC13239306; doi:10.1155/jare/6085101)
Supplement: Supplementary file 1 — Supporting Information The supporting information includes the PRISMA 2020 reporting checklist (Table S1) and the complete search strategies used across PUBMED/MEDLINE, Web of Science, and Cochrane databases (Table S2). [file JARE-2026-6085101-s001.docx]

**Supplementary material**

**Table S1. PRISMA 2020 Checklist**

| **Section and Topic** | **Item #** | **Checklist item** | **Reported on page** |
| --- | --- | --- | --- |
| **TITLE** | | |  |
| Title | 1 | Identify the report as a systematic review. | 1 |
| **ABSTRACT** | | |  |
| Abstract | 2 | See the PRISMA 2020 for Abstracts checklist. | 1 |
| **INTRODUCTION** | | |  |
| Rationale | 3 | Describe the rationale for the review in the context of existing knowledge. | 2-3 |
| Objectives | 4 | Provide an explicit statement of the objective(s) or question(s) the review addresses. | 3 |
| **METHODS** | | |  |
| Eligibility criteria | 5 | Specify the inclusion and exclusion criteria for the review and how studies were grouped for the syntheses. | 3 |
| Information sources | 6 | Specify all databases, registers, websites, organisations, reference lists and other sources searched or consulted to identify studies. Specify the date when each source was last searched or consulted. | 3-4 |
| Search strategy | 7 | Present the full search strategies for all databases, registers and websites, including any filters and limits used. | 3-4 |
| Selection process | 8 | Specify the methods used to decide whether a study met the inclusion criteria of the review, including how many reviewers screened each record and each report retrieved, whether they worked independently, and if applicable, details of automation tools used in the process. | 4 |
| Data collection process | 9 | Specify the methods used to collect data from reports, including how many reviewers collected data from each report, whether they worked independently, any processes for obtaining or confirming data from study investigators, and if applicable, details of automation tools used in the process. | 4 |
| Data items | 10a | List and define all outcomes for which data were sought. Specify whether all results that were compatible with each outcome domain in each study were sought (e.g. for all measures, time points, analyses), and if not, the methods used to decide which results to collect. | 4 |
|  | 10b | List and define all other variables for which data were sought (e.g. participant and intervention characteristics, funding sources). Describe any assumptions made about any missing or unclear information. | 4 |
| Study risk of bias assessment | 11 | Specify the methods used to assess risk of bias in the included studies, including details of the tool(s) used, how many reviewers assessed each study and whether they worked independently, and if applicable, details of automation tools used in the process. | 5 |
| Effect measures | 12 | Specify for each outcome the effect measure(s) (e.g. risk ratio, mean difference) used in the synthesis or presentation of results. | 5 |
| Synthesis methods | 13a | Describe the processes used to decide which studies were eligible for each synthesis (e.g. tabulating the study intervention characteristics and comparing against the planned groups for each synthesis (item #5)). | 5-6 |
|  | 13b | Describe any methods required to prepare the data for presentation or synthesis, such as handling of missing summary statistics, or data conversions. | 5-6 |
|  | 13c | Describe any methods used to tabulate or visually display results of individual studies and syntheses. | 5-6 |
|  | 13d | Describe any methods used to synthesize results and provide a rationale for the choice(s). If meta-analysis was performed, describe the model(s), method(s) to identify the presence and extent of statistical heterogeneity, and software package(s) used. | 6 |
|  | 13e | Describe any methods used to explore possible causes of heterogeneity among study results (e.g. subgroup analysis, meta-regression). | 6 |
|  | 13f | Describe any sensitivity analyses conducted to assess robustness of the synthesized results. | 6 |
| Reporting bias assessment | 14 | Describe any methods used to assess risk of bias due to missing results in a synthesis (arising from reporting biases). | 6 |
| Certainty assessment | 15 | Describe any methods used to assess certainty (or confidence) in the body of evidence for an outcome. | NA |
| **RESULTS** | | |  |
| Study selection | 16a | Describe the results of the search and selection process, from the number of records identified in the search to the number of studies included in the review, ideally using a flow diagram. | 6 |
|  | 16b | Cite studies that might appear to meet the inclusion criteria, but which were excluded, and explain why they were excluded. | 6 |
| Study characteristics | 17 | Cite each included study and present its characteristics. | 6 |
| Risk of bias in studies | 18 | Present assessments of risk of bias for each included study. | 7 |
| Results of individual studies | 19 | For all outcomes, present, for each study: (a) summary statistics for each group (where appropriate) and (b) an effect estimate and its precision (e.g. confidence/credible interval), ideally using structured tables or plots. | 7 |
| Results of syntheses | 20a | For each synthesis, briefly summarise the characteristics and risk of bias among contributing studies. | 7-8 |
|  | 20b | Present results of all statistical syntheses conducted. If meta-analysis was done, present for each the summary estimate and its precision (e.g. confidence/credible interval) and measures of statistical heterogeneity. If comparing groups, describe the direction of the effect. | 7-8 |
|  | 20c | Present results of all investigations of possible causes of heterogeneity among study results. | 8 |
|  | 20d | Present results of all sensitivity analyses conducted to assess the robustness of the synthesized results. | 8 |
| Reporting biases | 21 | Present assessments of risk of bias due to missing results (arising from reporting biases) for each synthesis assessed. | 7 |
| Certainty of evidence | 22 | Present assessments of certainty (or confidence) in the body of evidence for each outcome assessed. | N/A |
| **DISCUSSION** | | |  |
| Discussion | 23a | Provide a general interpretation of the results in the context of other evidence. | 20 |
|  | 23b | Discuss any limitations of the evidence included in the review. | 11-12 |
|  | 23c | Discuss any limitations of the review processes used. | 11-12 |
|  | 23d | Discuss implications of the results for practice, policy, and future research. | 12-13 |
| **OTHER INFORMATION** | | |  |
| Registration and protocol | 24a | Provide registration information for the review, including register name and registration number, or state that the review was not registered. | 3 |
|  | 24b | Indicate where the review protocol can be accessed, or state that a protocol was not prepared. | 3 |
|  | 24c | Describe and explain any amendments to information provided at registration or in the protocol. | NA |
| Support | 25 | Describe sources of financial or non-financial support for the review, and the role of the funders or sponsors in the review. | 2 |
| Competing interests | 26 | Declare any competing interests of review authors. | 2 |
| Availability of data, code and other materials | 27 | Report which of the following are publicly available and where they can be found: template data collection forms; data extracted from included studies; data used for all analyses; analytic code; any other materials used in the review. | 2 |

Not applicable (NA).

*From:*  Page MJ, McKenzie JE, Bossuyt PM, Boutron I, Hoffmann TC, Mulrow CD, et al. The PRISMA 2020 statement: an updated guideline for reporting systematic reviews. BMJ 2021;372:n71. doi: 10.1136/bmj.n71. This work is licensed under CC BY 4.0. To view a copy of this license, visit <https://creativecommons.org/licenses/by/4.0/>

**Table S2. Complete Search Strategy**

| Database | Full-search’s equation |
| --- | --- |
| PUBMED/MEDLINE (N= 1,261) | (((((((("Aged"[Mesh])) OR (Aged[Text Word])) OR (Aged, 80 and over OR Centenarians OR Nonagenarians OR Octogenarians OR Frail Elderly)) OR ((("Home Nursing"[Mesh]) OR (Home Nursing[Text Word])) OR (Home Care, Nonprofessional[Text Word] OR Care, Nonprofessional Home[Text Word] OR Nonprofessional Home Care[Text Word] OR Home Care, Non-Professional[Text Word] OR Care, Non-Professional Home[Text Word] OR Home Care, Non-Professional[Text Word] OR Non-Professional Home Care[Text Word] OR Nursing, Home[Text Word]))) OR ((("Institutionalization"[Mesh]) OR (Institutionalization[Text Word])) OR (Institutionalizations[Text Word] OR Institutionalized Persons[Text Word] OR Institutionalized Person[Text Word] OR Person, Institutionalized[Text Word] OR Persons, Institutionalized[Text Word]))) AND ((((("Wearable Electronic Devices"[Mesh]) OR (Wearable Electronic Devices[Text Word])) OR (Device, Wearable Electronic[Text Word] OR Electronic Device, Wearable[Text Word] OR Wearable Electronic Device[Text Word] OR Wearable Technology[Text Word] OR Technology, Wearable[Text Word] OR Wearable Technologies[Text Word] OR Wearable Devices[Text Word] OR Device, Wearable[Text Word] OR Wearable Device[Text Word] OR Electronic Skin[Text Word] OR Skin, Electronic[Text Word] OR Accelerometer[Text Word] OR Accelerometers[Text Word] OR Activity-based monitor[Text Word] OR Activity-based monitors[Text Word])) OR ((("Exercise"[Mesh]) OR (Exercise[Text Word])) OR (Exercises[Text Word] OR Physical Activity[Text Word] OR Activities, Physical[Text Word] OR Activity, Physical[Text Word] OR Physical Activities[Text Word] OR Exercise, Physical[Text Word] OR Exercises, Physical[Text Word] OR Physical Exercise[Text Word] OR Physical Exercises[Text Word] OR Acute Exercise[Text Word] OR Acute Exercises[Text Word] OR Exercise, Acute[Text Word] OR Exercises, Acute[Text Word] OR Exercise, Isometric[Text Word] OR Exercises, Isometric[Text Word] OR Isometric Exercises[Text Word] OR Isometric Exercise[Text Word] OR Exercise, Aerobic[Text Word] OR Aerobic Exercise[Text Word] OR Aerobic Exercises[Text Word] OR Exercises, Aerobic[Text Word] OR Exercise Training[Text Word] OR Exercise Trainings[Text Word] OR Training, Exercise[Text Word] OR Trainings, Exercise[Text Word]))) OR ((("Sedentary Behavior"[Mesh]) OR (Sedentary Behavior[Text Word])) OR (Behavior, Sedentary[Text Word] OR Sedentary Behaviors[Text Word] OR Sedentary Lifestyle[Text Word] OR Lifestyle, Sedentary[Text Word] OR Physical Inactivity[Text Word] OR Inactivity, Physical[Text Word] OR Lack of Physical Activity[Text Word] OR Sedentary Time[Text Word] OR Sedentary Times[Text Word] OR Time, Sedentary[Text Word])))) AND ((("Frailty"[Mesh]) OR (Frailty[Text Word])) OR (Frailties[Text Word] OR Frailness[Text Word] OR Frailty Syndrome[Text Word] OR Debility[Text Word] OR Debilities[Text Word]))) AND (((("Cross-Sectional Studies"[Mesh]) OR (Cross-Sectional Studies[Text Word])) OR (Cross Sectional Studies[Text Word] OR Cross-Sectional Study[Text Word] OR Studies, Cross-Sectional[Text Word] OR Study, Cross-Sectional[Text Word] OR Cross Sectional Analysis[Text Word] OR Analyses, Cross Sectional[Text Word] OR Cross Sectional Analyses[Text Word] OR Disease Frequency Surveys[Text Word] OR Cross-Sectional Survey[Text Word] OR Cross Sectional Survey[Text Word] OR Cross-Sectional Surveys[Text Word] OR Survey, Cross-Sectional[Text Word] OR Surveys, Cross-Sectional[Text Word] OR Surveys, Disease Frequency[Text Word] OR Disease Frequency Survey[Text Word] OR Survey, Disease Frequency[Text Word] OR Analysis, Cross-Sectional[Text Word] OR Analyses, Cross-Sectional[Text Word] OR Analysis, Cross Sectional[Text Word] OR Cross-Sectional Analyses[Text Word] OR Cross-Sectional Analysis[Text Word] OR Prevalence Studies[Text Word] OR Prevalence Study[Text Word] OR Studies, Prevalence[Text Word] OR Study, Prevalence[Text Word])) OR (((("Cohort Studies"[Mesh])) OR (Cohort Studies[Text Word])) OR (Cohort Study[Text Word] OR Studies, Cohort[Text Word] OR Study, Cohort[Text Word] OR Concurrent Studies[Text Word] OR Studies, Concurrent[Text Word] OR Concurrent Study[Text Word] OR Study, Concurrent[Text Word] OR Closed Cohort Studies[Text Word] OR Cohort Studies, Closed[Text Word] OR Closed Cohort Study[Text Word] OR Cohort Study, Closed[Text Word] OR Study, Closed Cohort[Text Word] OR Studies, Closed Cohort[Text Word] OR Birth Cohort Studies[Text Word] OR Birth Cohort Study[Text Word] OR Cohort Studies, Birth[Text Word] OR Cohort Study, Birth[Text Word] OR Studies, Birth Cohort[Text Word] OR Study, Birth Cohort[Text Word] OR Analysis, Cohort[Text Word] OR Analyses, Cohort[Text Word] OR Cohort Analyses[Text Word] OR Cohort Analysis[Text Word] OR Historical Cohort Studies[Text Word] OR Cohort Studies, Historical[Text Word] OR Cohort Study, Historical[Text Word] OR Historical Cohort Study[Text Word] OR Study, Historical Cohort[Text Word] OR Studies, Historical Cohort[Text Word] OR Incidence Studies[Text Word] OR Incidence Study[Text Word] OR Studies, Incidence[Text Word] OR Study, Incidence[Text Word]))) |
| WEB OF SCIENCE (N= 70) | **#1**    (((((((((((((((((((TS=(Aged)) OR TS=(Aged, 80 and over)) OR TS=(Centenarians)) OR TS=(Nonagenarians)) OR TS=(Octogenarians)) OR TS=(Frail Elderly)) OR TS=(Home Nursing)) OR TS=(Home Care, Nonprofessional)) OR TS=(Care, Nonprofessional Home)) OR TS=(Nonprofessional Home Care)) OR TS=(Home Care, Non-Professional)) OR TS=(Care, Non-Professional Home)) OR TS=(Home Care, Non-Professional)) OR TS=(Institutionalization)) OR TS=(Institutionalizations)) OR TS=(Institutionalized Persons)) OR TS=(Institutionalized Person)) OR TS=(Person, Institutionalized)) OR TS=(Persons, Institutionalized)  **#2**  (((((((((((((((((((((TS=(Wearable Electronic Devices)) OR TS=(Wearable Technology)) OR TS=(Wearable Technologies)) OR TS=(Wearable Devices)) OR TS=(Electronic Skin)) OR TS=(Accelerometer))) OR TS=(Accelerometers)) OR TS=(Activity-based monitor)) OR TS=(Activity-based monitors)) OR TS=(Exercise)) OR TS=(Physical Activity)) OR TS=(Physical Activities)) OR TS=(Energy expenditure)) OR TS=(Metabolism)) OR TS=(Sedentary Behavior)) OR TS=(Sedentary Behaviors)) OR TS=(Sedentary Lifestyle)) OR TS=(Physical Inactivity)) OR TS=(Lack of Physical Activity)) OR TS=(Sedentary Time)) OR TS=(Sedentary Times)  **#3**  ((((TS=(Frailty)) OR TS=(Frailties)) OR TS=(Frailness)) OR TS=(Frailty Syndrome)) OR TS=(Debility)  **#4**  (((((((((((((((((((((TS=(Wearable Electronic Devices)) OR TS=(Wearable Technology)) OR TS=(Wearable Technologies)) OR TS=(Wearable Devices)) OR TS=(Electronic Skin)) OR TS=(Accelerometer))) OR TS=(Accelerometers)) OR TS=(Activity-based monitor)) OR TS=(Activity-based monitors)) OR TS=(Exercise)) OR TS=(Physical Activity)) OR TS=(Physical Activities)) OR TS=(Energy expenditure)) OR TS=(Metabolism)) OR TS=(Sedentary Behavior)) OR TS=(Sedentary Behaviors)) OR TS=(Sedentary Lifestyle)) OR TS=(Physical Inactivity)) OR TS=(Lack of Physical Activity)) OR TS=(Sedentary Time)) OR TS=(Sedentary Times) |
| COCHRANE (N= 304) | #1  (Aged) OR (Aged, 80 and over) OR (Centenarians) OR (Nonagenarians) OR (Octogenarians) OR (Frail Elderly) OR (Home Nursing) OR (Home Nursing) OR (Home Care), (Nonprofessional) OR (Care, Nonprofessional Home) OR (Nonprofessional Home Care) OR (Home Care, Non-Professional) OR (Care, Non-Professional Home) OR (Home Care, Non-Professional) OR (Non-Professional Home Care) OR (Nursing, Home) OR (Institutionalization) OR (Institutionalization) OR (Institutionalizations) OR (Institutionalized Persons) OR (Institutionalized Person) OR (Person, Institutionalized) OR (Persons, Institutionalized)  #2  (Wearable Electronic Devices) OR (Wearable Electronic Devices) OR (Device, Wearable Electronic) OR (Electronic Device, Wearable) OR (Wearable Electronic Device) OR (Wearable Technology) OR (Technology, Wearable) OR (Wearable Technologies) OR (Wearable Devices) OR (Device, Wearable) OR (Wearable Device) OR (Electronic Skin) OR (Skin, Electronic) OR (Accelerometer) OR (Accelerometers) OR (Activity-based monitor) OR (Activity-based monitors) OR (Exercise) OR (Exercise) OR (Exercises) OR (Physical Activity) OR (Activities, Physical) OR (Activity, Physical) OR (Physical Activities) OR (Exercise, Physical) OR (Exercises, Physical) OR (Physical Exercise) OR (Physical Exercises) OR (Sedentary Behavior) OR (Sedentary Behavior) OR (Behavior, Sedentary) OR (Sedentary Behaviors) OR (Sedentary Lifestyle) OR (Lifestyle, Sedentary) OR (Physical Inactivity) OR (Inactivity, Physical) OR (Lack of Physical Activity) OR (Sedentary Time) OR (Sedentary Times) OR (Time, Sedentary)  #3  (Frailty) OR (Frailties) OR (Frailness) OR (Frailty Syndrome) OR (Debility) OR (Debilities)  #4  (Cross-Sectional Studies) OR (Cross Sectional Studies) OR (Cross-Sectional Study) OR (Studies, Cross-Sectional) OR (Study, Cross-Sectional) OR (Cross Sectional Analysis) OR (Analyses, Cross Sectional) OR (Cross Sectional Analyses) OR (Disease Frequency Surveys) OR (Cross-Sectional Survey) OR (Cross Sectional Survey) OR (Cross-Sectional Surveys) OR (Survey, Cross-Sectional) OR (Surveys, Cross-Sectional) OR (Surveys, Disease Frequency) OR (Disease Frequency Survey) OR (Survey, Disease Frequency) OR (Analysis, Cross-Sectional) OR (Analyses, Cross-Sectional) OR (Analysis, Cross Sectional) OR (Cross-Sectional Analyses) OR (Cross-Sectional Analysis) OR (Prevalence Studies) OR (Prevalence Study) OR (Studies, Prevalence) OR (Study, Prevalence) OR (Cohort Studies) OR (Cohort Study) OR (Studies, Cohort) OR (Study, Cohort) OR (Concurrent Studies) OR (Studies, Concurrent) OR (Concurrent Study) OR (Study, Concurrent) OR (Closed Cohort Studies) OR (Cohort Studies, Closed) OR (Closed Cohort Study) OR (Cohort Study, Closed) OR (Study, Closed Cohort) OR (Studies, Closed Cohort) OR (Birth Cohort Studies) OR (Birth Cohort Study) OR (Cohort Studies, Birth) OR (Cohort Study, Birth) OR (Studies, Birth Cohort) OR (Study, Birth Cohort) OR (Analysis, Cohort) OR (Analyses, Cohort) OR (Cohort Analyses) OR (Cohort Analysis) OR (Historical Cohort Studies) OR (Cohort Studies, Historical) OR (Cohort Study, Historical) OR (Historical Cohort Study) OR (Study, Historical Cohort) OR (Studies, Historical Cohort) OR (Incidence Studies) OR (Incidence Study) OR (Studies, Incidence) OR (Study, Incidence)  #1 AND #2 AND #3 AND #4 |
